# Using Inertial and Physiological Sensors to Investigate the Effects of a High-Intensity Interval Training and Plyometric Program on the Performance of Young Judokas

**DOI:** 10.3390/s22228759

**Published:** 2022-11-12

**Authors:** Adrián Mañas-Paris, José M. Muyor, José M. Oliva-Lozano

**Affiliations:** 1 Department of Education, University of Almería, 04120 Almería, Spain; 2 Laboratory of Kinesiology, Biomechanics and Ergonomics (KIBIOMER Lab.), Research Central Services, University of Almería, 04120 Almería, Spain; 3 Health Research Centre, University of Almería, 04120 Almería, Spain

**Keywords:** motion capture, motion analysis, physical activity sensing, wireless sensor networks, monitoring application

## Abstract

The use of inertial and physiological sensors in a sport such as judo is scarce to date. The information provided by these sensors would allow practitioners to have a better understanding of sports performance, which is necessary for an accurate training prescription. The purpose of this study was to use inertial and physiological sensors in order to investigate the effect of a plyometric and high-intensity interval training (HIIT) training program on Special Judo Fitness Test (SJFT) performance and speed of execution of throws in young judokas. A total of 32 participants were divided into two groups: experimental and control. The intervention consisted of six sessions with a duration of 60 min for 3 weeks. Physiological sensors collected heart rate data to assess the Special Judo Fitness Test, and inertial sensors collected angular velocity. The results show a significant decrease in the SJFT index (Score pre: 22.27 ± 2.73; Score post: 19.65 ± 1.70; *p* ≤ 0.05; *d* = 0.61) and a significant increase in the angular velocity of the *X*-axis (Pre: 320.87 ± 51.15°/s; Post: 356.50 ± 40.47°/s; *p* ≤ 0.05; *d* = 0.45) and *Y*-axis (Pre: 259.40 ± 41.99°/s; Post: 288.02 ± 65.12°/s; *p* ≤ 0.05; *d* = 0.31) in the experimental group. In conclusion, this study demonstrates that using inertial and physiological sensors allowed us to analyze the effect that a high-intensity interval training program and plyometrics had on the performance of young judokas. Strength and conditioning coaches should consider these results because including plyometric training and HIIT in judokas’ workout programming can be especially positive for eliciting increases in performance. However, future training interventions should investigate the training adaptations to longer interventions.

## 1. Introduction

In recent years, an increasing number of publications have applied inertial sensors technology to quantify physical activity and sports performance [1,2,3]. The main advantage of inertial sensors is that they may be used in the field context, and a substantial amount of performance external load measures may be collected [3,4]. Moreover, some inertial sensors may synchronize with physiological sensors that collect internal load measures (e.g., heart rate) [5]. Consequently, this allows practitioners to obtain a better understanding of sports performance, and several systems are currently available on the market. 

Judo is a Japanese martial art in which players use body weight and balance to throw or pin their opponent [6]. It has been an Olympic sport since 1964 for the men’s category and since 1992 for the women’s category [7]. It is a high-intensity intermittent combat sport that requires complex skills, in which a wide variety of physical qualities must be developed to achieve optimal technical–tactical performance and, in turn, sporting success [8]. The competition is divided according to age and weight. The duration of the match is up to 5 min, but if an athlete obtains an Ippon (full point) the match ends regardless of the remaining time. In the event that there is no winner, the match is decided on golden score. Therefore, the duration can range from a few seconds to golden score [9].

The practice of judo during childhood and adolescence is related to several beneficial effects on the parameters of cardiovascular and bone health [10]. As observed in a recent study [11], it is established that physical activity benefits executive functions and academic performance in children. Results show that children who practiced martial arts presented better executive functioning and higher school grades [12]. These findings affirm that judo is an effective exercise for health promotion and an optimal way to maintain physical activity and practice sports [13]. As a consequence of the numerous benefits developed above, achieving greater sports performance in judo can increase sports motivation. Performance can be evaluated using the validated SJFT, and its application in this population lacks studies regarding performance and physiological adaptations [14].

The SJFT [15] is a popular and specific test for judo since it aims to assess the aerobic and anaerobic lactic capacity of the judoka. This test, of an intermittent nature, is especially helpful and has provided a significant improvement in the study of judo since it provides an advantage by recreating real movements in the discipline, specifically the Ippon-Seoi-Nage technique [16]. As explained above, the duration of a judo match is not exact. However, most matches typically last 3–4 min [17], with 20–30 s periods of activity and 5–10 s of breaks or pauses [18]. Therefore, the duration of the test is related to the time and the real energy demands of a fight. Furthermore, according to a recent study [19], the importance of execution speed in projections in the SJFT has been observed. In the training process, physical fitness tests, special fitness tests specific to the sport, and kinematic measures should be used to give optimal control to training [20,21]. In addition, the kinematic variables play a fundamental role since judo is a very technical sport, and the moment of application of force and movement (performing a multitude of turns) are decisive [22]. Therefore, a relationship is observed between the performance and analysis from the point of view of the rotation in the projections.

Although there are studies in young athletes from other sports (e.g., baseball or swimming) analyzing biomechanics and physiological responses [23,24], there is currently a lack of studies in the literature on the application of inertial and physiological sensors technology to analyze sports performance in judokas, specifically in children and adolescents [25]. As observed in a previous study [26], it has only been investigated in adult judokas. However, coaches may use this information to design training strategies and optimize performance. For example, one might wonder which training strategies enhance performance in the SJFT. Regarding the specific training protocol for judokas, there is no isolated training system, and they can be subjected to several mixed protocols [27]: standard judo training with strength training, standard judo training with aerobic training, and standard judo training with HIIT [28]. However, plyometric training with HIIT seems to be the best combination to improve both the result of the SJFT [29,30,31] and neuromechanical adaptations [32]. 

Therefore, the main objective of this study was to use inertial and physiological sensors in order to investigate the effect of a plyometric and HIIT training program on SJFT performance and the speed of execution of throws in young judokas. Furthermore, since the SJFT is composed of three rounds of projections, the effect of the round on the execution speed of the projections was considered.

## 2. Materials and Methods

### 2.1. Study Design

This study was designed as a non-randomized experimental intervention, based on organizational criteria derived from practical principles. The sample was divided into 2 groups. The experimental and control groups were measured during the SJFT before and after the intervention period for 3 weeks. During this period, both groups completed their regular 3 sessions of standard judo training (duration per session: 90 min) per week. However, the experimental group performed 2 sessions of plyometric training combined with HIIT (duration per session: 60 min) per week.

### 2.2. Participants

**Figure 1:**
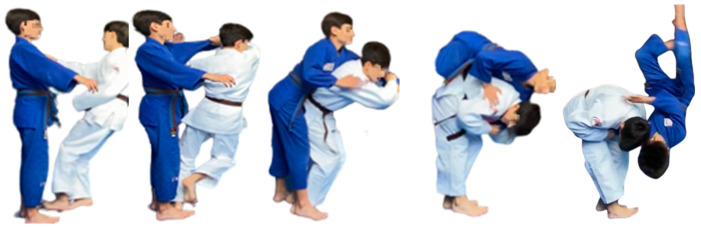
Ippon-Seoi-Nage technique.

Thirty-two participants (age: 12.84 ± 1.69 years; height: 157.19 ± 12.59 cm; weight: 47.13 ± 14.01 kg; time practicing judo: 5.44 ± 1.87 years) voluntarily collaborated in the study. They were divided into two groups (experimental group: *n* = 16; control group: *n* = 16; both groups formed by 7 females and 9 males), organized regarding their usual activity schedule, taking into account that the sample was the same number of male and female participants for both groups. Participants were included if (a) they were born between 2007 and 2011, and (b) they knew how to perform the Ippon-Seoi-Nage throwing technique (Figure 1). If any judoka had experienced any musculoskeletal injury within the last 3 months before the study, they were excluded from the study. Before participation, they were informed of the performance of the test and the requirements and risks involved in the study. The consent of the father, mother, or legal guardian was requested since all were under 18 years of age. This study was conducted according to the Declaration of Helsinki, and it was approved by the University of Almeria Bioethics Committee (UALBIO2014/009).

### 2.3. Procedures

The data collection was carried out during the regular practice of their training. The SJFT was performed in all participants. A protocol was adapted for the entire sample, based on a previous study [33] (Table 1) performing the usual joint mobility of the sample training sessions and a slight individual plyometric activation prior to the test [29]. All the ukes (that is, subjects on whom the action is performed) were of similar weight and height to tori (the subject who performs the action and the test). The total number of throws completed by the toris during each of the three periods was recorded; the toris’ heart rate was immediately measured after the end of the test and 1 min after the test. The SJFT index was calculated according to the following equation: Index = (HR after + HR 1 min later)/total number of throws. The index value decreases with better test performance [34]. During the minute of recovery, they had to perform at the same mark where the test began. Specifically, the study’s variables were: the score obtained during the SJFT (Formula (1)) and the angular velocity in the sagittal and transverse planes during each projection of the SJFT. Formula (1): Calculation of the score obtained in the SJFT (end of the test + 1 min at the end of the test):

(1) $$\mathrm{SJFT}\;\mathrm{score}=\frac{\mathrm{HR}\;\mathrm{after}+\mathrm{HR}\;1\min\;\mathrm{later}}{\Sigma \;\mathrm{Throws}}$$

### 2.4. Instruments

The SJFT consists of performing the largest possible number of projections on two ukes located 6 m apart from each other, starting from an equidistant position, in three periods of time (15″ 30″ and 30″ with 10″ of passive recovery between each period). At the end, there is a passive rest of 1 min [16].

**Figure 2:**
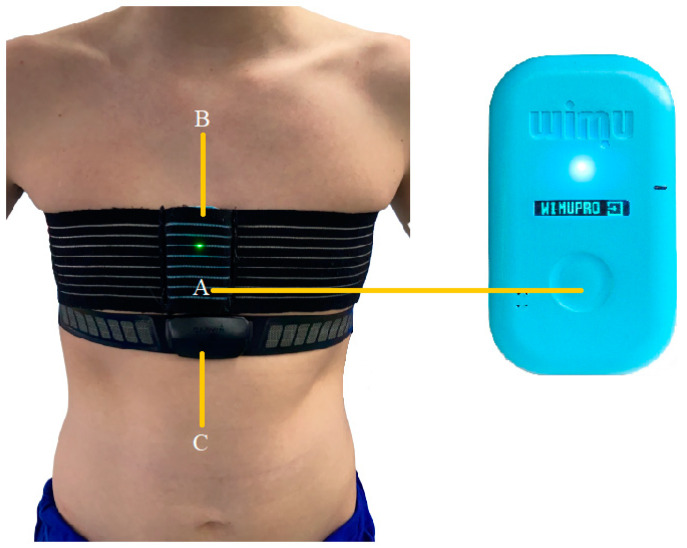
Tracking system (**A**) placed vertically in an elastic pocket (**B**) and the band to record the heart rate signal (**C**).

**Figure 3:**
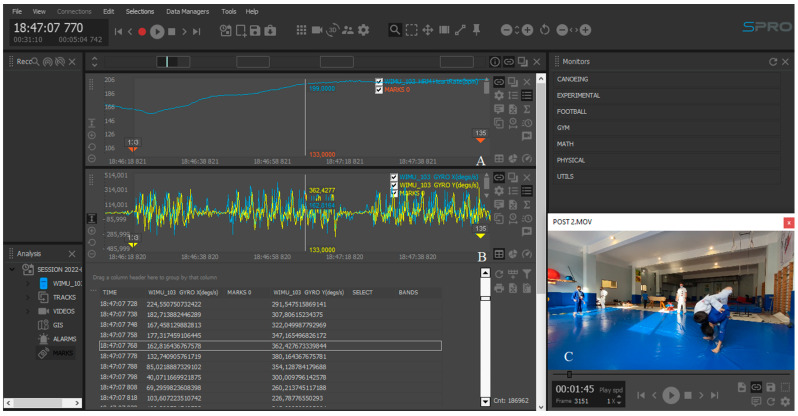
Data analysis on SPro software with different channels (A: heart rate; B: *X* and *Y*-axis angular speed; C: video synchronized with the data).

For the dynamic measurement of the trunk, a WIMU Pro device (RealTrack Systems, Almería, Spain) (Figure 2A) was used. This device consists of several inertial sensors (four 3D accelerometers, three 3D gyroscopes, a magnetometer, and a barometer) that collect data at a sampling frequency of 100 Hz [4]. Specifically, the device provides 3D (x, y, and z) angular velocity data. The device was placed vertically in an elastic pocket (Aptonia, Lille, France) [1] attached above the xiphoid process (Figure 2B). The device was calibrated just before the start of the test following the manufacturer’s instructions on WIMUNET (RealTrack Systems, Almeria, Spain). The device was then placed on a stable surface. The device was turned on and allowed a 30 s pause before starting to record the session. To record the heart rate signal, a GARMIN band (Garmin Ltd., Olathe, KS, USA) (Figure 2C) was used, which sent the data to the WIMU PRO system (RealTrack Systems, Almería, Spain) through Ant + technology with a sampling frequency of 4 Hz [4]. Once the data collection session finished, the data were transferred to SPro software (RealTrack Systems, Almería, Spain) and synchronized with the video (Figure 3).

### 2.5. Statistical Analysis

First, a Shapiro–Wilk normality test was performed to analyze the normality of the variables. Since the variables had a normal distribution, the paired Student‘s *t*-test was used to compare the data obtained in the pre-test and post-test performance variables. Between-group effect size (Cohen’s d) was calculated using a pooled standard deviation and classified as: trivial (0–0.19), small (0.20–0.49), medium (0.50–0.79), and large (≥0.8) [35]. In addition, in order to determine the effect each round of the SJFT had on the angular velocity in each axis of movement and group of participants, a general linear model of repeated measures was performed. Statistical analysis was performed using IBM SPSS Statistics version 25 software (SPSS, Inc., Armonk, NY, USA), and the level of significance was set at *p* ≤ 0.05.

## 3. Results

Table 2 shows the comparison between the result of the SJFT in the experimental group and control group. A decrease in the SJFT score with statistically significant differences (*p* ≤ 0.001) was observed in the experimental group. However, no significant changes were observed in the control group (*p* = 0.05).

Table 3 shows the comparison in the speed of rotation on the *X*- and *Y*-axes. An increase in the angular speed on the *X*-axis was observed with statistically significant differences (*p* ≤ 0.001) in the experimental group and significant differences on the *Y*-axis (*p* = 0.02). However, no significant changes were observed in the control group on the *X*-axis (*p* = 0.29), nor on the *Y*-axis (*p* = 0.92).

The angular velocity recorded in each axis of movement according to the rounds of the SJFT and the group of participants is shown below (Table 4). A significant effect of the “round variable’’ was observed in the angular velocity on the *X*-axis of the experimental group (F = 10.81; *p* ≤ 0.001; ηp^2^ = 0.68). Specifically, an increase in the angular velocity on the *X*-axis was observed with statistically significant differences (*p* = 0.01) in rounds two and three, in the experimental group. However, no significant changes were observed in the control group on the *X*-axis (*p* > 0.05) in any of the three rounds.

When it comes to the *Y*-axis, no significant differences (*p* > 0.05) were observed in the pairwise comparison for any of the rounds of SJFT or groups.

## 4. Discussion

The purpose of this study was to use inertial and physiological sensors to investigate the effect of a plyometric and HIIT training program on SJFT performance and execution speed of throws in young judokas. The main findings were that physiological sensors showed a significant decrease in the SJFT index, which implies an improvement in the test since the lower the index value the better the test performance [34], and inertial sensors showed a significant increase in angular velocity (both *X*- and *Y*-axes) in the experimental group. Furthermore, since the SJFT is composed of three rounds of projections, the effect of the round on the execution speed of the projections was considered.

The results of the intervention were satisfactory, obtaining a decrease in the score in the SJFT test in the experimental group (*p* ≤ 0.001) compared to the control group (*p* = 0.05). This is explained by the fact that the decrease in the index of the SJFT index is related to an increase in performance [34]. Consequently, three sessions of standard judo training per week combined with two sessions of plyometric training and HIIT per week may be considered a potential training strategy to improve the performance of young judokas. The use of physiological sensors is necessary because it is of great importance to monitor training adaptations [36] and assess physiological qualities for movement optimization [37]. The impact of fatigue has been observed as a key factor in performance and biomechanical variables [38], increasing the risk of injury with increased physiological fatigue, as it appears to be a multifaceted phenomenon involving central and peripheral factors, resulting in a slowing of the motor unit and a decrease in maximal force and power [39,40,41,42].

Regarding the rotation speed in the *X*- and *Y*-axes, significant increases in the angular speed in both axes were observed in the experimental group: (*p* ≤ 0.001) and (*p* = 0.02), respectively. Therefore, a significant improvement in the angular velocity and performance in the SJFT of the experimental group was observed in comparison with the control group. However, in the “round variable”, the only significant changes were seen in the *X*-axis for the experimental group (*p* = 0.01). In this axis, the movements are made in the transverse plane [43] and, in the *Y*-axis, the movements are made in the sagittal plane. Although most of the actions in the competition should be performed at maximum speed, it is important to study the kinematics of the actions, acquiring energy and technical efficiency, both for performance and injury prevention [44,45]. A biomechanical analysis can help to understand the techniques of a highly technical sport such as judo [46]. During the SJFT, increased mechanical work during periods of active throwing would affect performance through altered patterns of recovery and fatigue throughout the testing procedures [47]. Thus, a finding emerges regarding forward-looking performance variables [48], and the key factors that lead to the successful movement will help us to understand some unknown affordance of movement [49] due to the improvement of inertial sensors that have facilitated the evaluation of angular velocity and physiological variables.

Both groups performed standard judo training according to their regular annual training plan. Although the SJFT index and angular speeds did not significantly change in the control group, the mean values show a slight improvement in the control group (change in SJFT index: −0.39; change in *Y*-axis angular speed, round 1: 10.94°/s; change in *Y*-axis angular speed, round 2: 20.36°/s). This may be due to the possible improvement in the techniques performed in the standard judo training (e.g., the Ippon-Seoi-Nage technique was frequently included in their sessions) or familiarization with the test. However, the Ippon-Seoi-Nage technique is a complex action and its performance requires a learning process [50]. Only the experimental group significantly improved performance in the test, which is another clear argument highlighting the validity of the intervention program.

One of the advantages of this study is the novel application of non-invasive, time-resolved sensor data [51], supplemented with a heart rate monitor to record heart rate response. This may support the development of strategies to improve judo performance. Moreover, the development of technology has allowed us to obtain kinematics data, demonstrating that sports biomechanics is of interest to determine the mechanical power of certain movements [52]. The application of inertial and physiological sensors allowed us to analyze the impact that a plyometric training program combined with HIIT has on the performance of young judokas. In addition, another advantage on this study is that the study population is novel in the analysis of both biomechanics and heart response to SJFT [53,54], and that most of the literature has focused on injuries [7,44,55].

However, this study has some limitations. On the one hand, despite the fact that some studies have been published on the kinematics of judo [56,57], due to the technical limitations and the measurement environment during this period, research mainly focuses on investigating the static balance during the preparation phase in the throwing techniques [46], for which the variables of performance have inertial sensors. The sample could be larger, and the intervention time could be longer to assess whether performance keeps increasing. Moreover, conducting this kind of research on elite judokas could be more reliable since children and adolescents experience continuous growth and development [25]. In addition, the participants must be the same weight, height, and level, since two extra people are needed to carry out the test, who receive numerous impacts. Moreover, difficulties have been found in the fixation of the elastic pocket in the xiphoid process for female participants.

## 5. Conclusions

This study demonstrated that using inertial and physiological sensors allowed us to analyze the effect that a high-intensity interval training program and plyometrics combined with the standard judo training had on the performance of young judokas. The main findings of this study are that a significant improvement in the SJFT index and angular velocity (both *X*- and *Y*-axes) was observed in the experimental group. Therefore, this technology is important for measuring judo training performance. Strength and conditioning coaches should consider these results because including plyometric training and HIIT in judokas’ workout programming can be especially positive to elicit increases in performance.

## Figures and Tables

**Table 1:** Warm-up protocol adapted [33].

Exercise	Volume
Jogging	5 min
Stretching	5 min
Break fall drills (Front, back, side, and rolling)	5 repetitions each
Uchikomi (throwing drills)	1 × 10 repetitions
Rapid Uchikomi Ippon-Seoi-Nage	1 × 10 repetitions
Power Uchikomis Ippon-Seoi-Nage	1 × 5 repetitions
Nagekomi (practice throw)	1 × 5 repetitions
Vertical jump (before individual test)	2 × 4 repetitions
Horizontal jump (before individual test)	1 × 4 repetitions

**Table 2:** Comparison between pre-test and post-test evaluations in the SJFT.

Group	Variable	Pre-Test (Mean ± SD)	Post-Test (Mean ± SD)	Mean Differences (Mean ± SD)	*p*	*d* by Cohen
Experimental	SJFT (score)	22.27 ± 2.73	19.65 ± 1.70	2.61 ± 2.01	0.00	0.61
Control		20.66 ± 2.09	20.27 ± 2.69	0.3878 ± 2.43	0.53	0.00

Note: SJFT = Special Judo Fitness Test; SD = standard deviation.

**Table 3:** Comparison of the speed of rotation on the *X*- and *Y*-axes between pre-test and post-test evaluations in the SJFT.

Group	Variable	Pre-Test (Mean ± SD)	Post-Test (Mean ± SD)	Mean Differences (Mean ± SD)	*p*	*d* by Cohen
Experimental	Angular velocity on the *X*-axis (^o^/s)	320.87 ± 51.15	356.50 ± 40.47	35.62 ± 31.98	0.00	0.45
	Angular velocity on the *Y*-axis (^o^/s)	259.40 ± 41.99	288.02 ± 65.12	28.62 ± 44.71	0.02	0.31
Control	Angular velocity on the *X*-axis (^o^/s)	303.26 ± 43.85	297.71 ± 38.10	5.54 ± 20.28	0.29	0.00
	Angular velocity on the *Y*-axis (^o^/s)	241.33 ± 44.69	242.56 ± 36.65	1.23 ± 49.16	0.92	0.00

Note: SJFT = Special Judo Fitness Test; SD = standard deviation.

**Table 4:** Comparison of the differences in mean speed of rotation on each axis in the 3 rounds, between pre-test and post-test evaluations in the SJFT.

Axis	Group	Variable	Pre-Test (Mean ± SD)	Post-Test (Mean ± SD)	Mean Differences (Mean ± SD)	*p*	*d* by Cohen
*X*	Experimental	Angular velocity (^o^/s): R1	333.24 ± 62.33	368.66 ± 47.98	35.42 ± 11.71	0.08	0.30
		Angular velocity (^o^/s): R2	320.83 ± 56.95	355.59 ± 43.89	34.76 ± 9.22	0.01	0.40
		Angular velocity (^o^/s): R3	308.54 ± 46.18	345.23 ± 48.10	36.69 ± 9.59	0.01	0.31
	Control	Angular velocity (^o^/s): R1	313.55 ± 51.16	308.14 ± 51.64	5.41 ± 11.71	1.00	0.00
		Angular velocity (^o^/s): R2	300.01 ± 50.53	298.06 ± 33.59	1.95 ± 9.22	1.00	0.00
		Angular velocity (^o^/s): R3	296.20 ± 46.48	286.95 ± 36.71	9.26 ± 9.59	1.00	0.00
*Y*	Experimental	Angular velocity (^o^/s): R1	271.40 ± 53.69	303.50 ± 53.69	32.09 ± 14.95	0.60	0.36
		Angular velocity (^o^/s): R2	262.17 ± 50.83	281.19 ± 68.13	19.02 ± 12.71	1.00	0.10
		Angular velocity (^o^/s): R3	244.63 ± 38.39	279.39 ± 66.41	34.75 ± 14.30	0.32	0.31
	Control	Angular velocity (^o^/s): R1	234.43 ± 60.22	245.37 ± 49.33	10.94 ± 14.95	1.00	0.00
		Angular velocity (^o^/s): R2	249.22 ± 46.00	240.30 ± 36.83	8.93 ± 12.71	1.00	0.00
		Angular velocity (^o^/s): R3	240.35 ± 41.47	260.71 ± 55.97	1.68 ± 14.30	1.00	0.00

Note: SJFT = Special Judo Fitness Test; SD = standard deviation; R1 = Round 1; R2 = Round 2; R3 = Round 3.

## References

[1] Oliva-Lozano J.M., Martín-Fuentes I., Muyor J.M. (2020). Validity and reliability of a new inertial device for monitoring range of motion at the pelvis during sexual intercourse. Int. J. Environ. Res. Public Health.

[2] Poitras I., Dupuis F., Bielmann M., Campeau-Lecours A., Mercier C., Bouyer L.J., Roy J.S. (2019). Validity and reliability of wearable sensors for joint angle estimation: A systematic review. Sensors.

[3] Oliva-Lozano J.M., Maraver E.F., Fortes V., Muyor J.M. (2020). Kinematic analysis of the postural demands in professional soccer match play using inertial measurement units. Sensors.

[4] Oliva-Lozano J.M., Martín-Fuentes I., Muyor J.M. (2020). Validity and reliability of an inertial device for measuring dynamic weight-bearing ankle dorsiflexion. Sensors.

[5] Gómez-Carmona C.D., Bastida-Castillo A., García-Rubio J., Ibáñez S.J., Pino-Ortega J. (2018). Static and dynamic reliability of WIMU PRO^TM^ accelerometers according to anatomical placement. J. Sports Eng. Technol..

[6] Bu B., Haijun H., Yong L., Chaohui Z., Xiaoyuan Y., Singh M.F. (2010). Effects of martial arts on health status: A systematic review. J. Evid. Based Med..

[7] Pocecco E., Ruedl G., Stankovic N., Sterkowicz S., del Vecchio F.B., Gutiérrez-García C., Rousseau R., Wolf M., Kopp M., Miarka B. (2013). Injuries in judo: A systematic literature review including suggestions for prevention. Br. J. Sports Med..

[8] Soto D., Aedo-Muñoz E., José Brito C., Miarka B. (2020). Comparisons of motor actions and biomechanical assessments of judo techniques between female weight categories. J. Hum. Kinet..

[9] Franchini E., del Vecchio F.B., Matsushigue K.A., Artioli G.G. (2011). Physiological profiles of elite judo athletes. Sports Med..

[10] Suetake V.Y.B., Franchini E., Saraiva B.T.C., da Silva A.K.F., Bernardo A.F.B., Gomes R.L., Vanderlei L.C.M., Christofaro D.G.D. (2018). Effects of 9 months of martial arts training on cardiac autonomic modulation in healthy children and adolescents. Pediatr. Exerc. Sci..

[11] Giordano G., Gómez-López M., Alesi M. (2021). Sports, Executive functions and academic performance: A comparison between martial arts, team sports, and sedentary children. Int. J. Environ. Res. Public Health.

[12] Becker D.R., McClelland M.M., Geldhof G.J., Gunter K.B., MacDonald M. (2018). Open-skilled sport, sport intensity, executive function, and academic achievement in grade school children. Early Educ. Dev..

[13] Milligan K., Cosme R., Wolfe Miscio M., Mintz L., Hamilton L., Cox M., Woon S., Gage M., Phillips M. (2017). Integrating mindfulness into mixed martial arts training to enhance academic, social, and emotional outcomes for at-risk high school students: A qualitative exploration. Contemp. Sch. Psychol..

[14] Rodríguez L., Prieto Saborit J.A., González Díez V. (2012). Descripción de diversos test para la valoración de la condición física en judo. Rev. De Artes Marciales Asiáticas.

[15] Sterkowicz S., Franchini E. (1995). The special judo fitness test. Antropomotoryka.

[16] Rodríguez C., Hernández-García R., Robles C., Torres-Luque G. (2016). Validación del special judo fitness test con la técnica tokui waza. Estudio piloto. SPORT TK Rev. EuroAm. Cienc. Deporte.

[17] Castanerlas J.L., Planas A. (1997). Study of temporal structure in judo contest. Apunts. Educ. Física Deportes.

[18] Van Malderen K., Jacobs C., Ramon K., Zinzen E., Deriemaeker P., Clarys P. (2009). Time and technique analysis of a judo fight: A comparison between males and females. Book of Abstracts of the 11th Annual Congress of the European College of Sport Sciences.

[19] Sterkowicz-Przybycień K., Fukuda D.H., Franchini E. (2019). Meta-analysis to determine normative values for the special judo fitness test in male athletes: 20+ years of sport-specific data and the lasting legacy of stanisław sterkowicz. Sports.

[20] Boguszewska K., Boguszewski D., Buśko K. (2010). Special judo fitness test and biomechanics measurements as a way to control of physical fitness in young judoists. Arch. Budo Sci. Martial Arts..

[21] Katralli J., Goudar S.S. (2012). Anthropometric profile and special judo fitness levels of indian judo players. Asian J. Sports Med..

[22] Imamura R.T., Hreljac A., Escamilla R.F., Edwards W.B. (2006). A three-dimensional analysis of the center of mass for three different judo throwing techniques. J. Sports Sci. Med..

[23] Okoroha K.R., Lizzio V.A., Meta F., Ahmad C.S., Moutzouros V., Makhni E.C. (2018). Predictors of elbow torque among youth and adolescent baseball pitchers. Am. J. Sports Med..

[24] Morais J.E., Barbosa T.M., Forte P., Silva A.J., Marinho D.A. (2021). Young swimmers’ anthropometrics, biomechanics, energetics, and efficiency as underlying performance factors: A systematic narrative review. Front. Physiol..

[25] Kons R.L., da Silva Athayde M.S., da Silva Junior J.N., Katcipis L.F.G., Detanico D. (2020). Predictors of judo-specific tasks from neuromuscular performance in young athletes aged 11–16 years. Int. J. Sports Phys. Ther..

[26] Detanico D., Dal Pupo J., Franchini E., Giovana dos Santos S. (2012). Relationship of aerobic and neuromuscular indexes with specific actions in judo. Sci. Sports.

[27] Da Silva L., Neto N., Lopes-Silva J., Leandro C., Silva-Cavalcante M. (2021). Training protocols and specific performance in judo athletes: A systematic review. J. Strength Cond. Res..

[28] Lopes-Silva J.P., Panissa V.L.G., Julio U.F., Franchini E. (2021). Influence of physical fitness on special judo fitness test performance: A multiple linear regression analysis. J. Strength Cond. Res..

[29] Miarka B., del Vecchio F.B., Franchini E. (2011). Acute effects and postactivation potentiation in the special judo fitness test. J. Strength Cond. Res..

[30] Franchini E., Julio U.F., Gonçalves Panissa V.L., Lira F.S., Agostinho M.F., Branco B.H.M. (2017). Short-term low-volume high-intensity intermittent training improves judo-specific performance. J. Sci. Med. Sport.

[31] Sole S., Ramírez-Campillo R., Andrade D.C., Sanchez-Sanchez J. (2021). Plyometric jump training effects on the physical fitness of individual-sport athletes: A systematic review with meta-analysis. PeerJ.

[32] Versteegh T.H., Dickey J.P., Emery C.A., Fischer L.K., Macdermid J.C., Walton D.M. (2020). Evaluating the effects of a novel neuromuscular neck training device on multiplanar static and dynamic neck strength: A pilot study. J. Strength Cond. Res..

[33] Lum D. (2019). Effects of various warm-up protocol on special judo fitness test performance. J. Strength Cond. Res..

[34] Casals C., Huertas J.R., Franchini E., Sterkowicz-Przybycién K., Sterkowicz S., Gutiérrez-García C., Escobar-Molina R. (2017). Special judo fitness test level and anthropometric profile of elite spanish judo athletes. J. Strength Cond. Res..

[35] Cohen J. (2013). Statistical Power Analysis for the Behavioral Sciences.

[36] Meeusen R., Duclos M., Foster C., Fry A., Gleeson M., Nieman D., Raglin J., Rietjens G., Steinacker J., Urhausen A. (2012). Prevention, diagnosis and treatment of the overtraining syndrome: Joint consensus statement of the european college of sport science (ECSS) and the american college of sports medicine (ACSM). Eur. J. Sport Sci..

[37] Bridge C.A., Ferreira Da Silva Santos J., Chaabène H., Pieter W., Franchini E. (2014). Physical and physiological profiles of taekwondo athletes. Sports Med..

[38] Aquino M., Petrizzo J., Otto R.M., Wygand J. (2022). The impact of fatigue on performance and biomechanical variables—A narrative review with prospective methodology. Biomechanics.

[39] Ament W., Verkerke G. (2012). Exercise and fatigue. Sports Med..

[40] Li F., Rupčić T., Knjaz D. (2021). The effect of fatigue on kinematics and kinetics of basketball dribbling with changes of direction. Kinesiology.

[41] Belcic I., Rodić S., Dukarić V., Rupčić T., Knjaz D. (2021). Do blood lactate levels affect the kinematic patterns of jump shots in handball?. Int. J. Environ. Res. Public Health.

[42] Sant’Ana J., Franchini E., da Silva V., Diefenthaeler F. (2016). Effect of fatigue on reaction time, response time, performance time, and kick impact in taekwondo roundhouse kick. Sports Biomech..

[43] Wang X., Chen Q., Wang W. (2014). 3D human motion editing and synthesis: A survey. Comput. Math. Methods Med..

[44] Koshida S., Ishii T., Matsuda T., Hashimoto T. (2017). Kinematics of judo breakfall for osoto-gari: Considerations for head injury prevention. J. Sports Sci..

[45] Vacca L., Rosso V., Gastaldi L. (2020). Risk assessment in different judo techniques for children and adolescent athletes. Proc. Inst. Mech. Eng. H..

[46] Ishii T., Ae M., Suzuki Y., Kobayashi Y. (2018). Kinematic comparison of the seoi-nage judo technique between elite and college athletes. Sports Biomech..

[47] Sterkowicz-Przybycień K.L., Fukuda D.H. (2014). Establishing normative data for the special judo fitness test in female athletes using systematic review and meta-analysis. J. Strength Cond. Res..

[48] Giudicelli B.B., Luz L.G.O., Sogut M., Massart A.G., Júnior A.C., Figueiredo A.J. (2020). Bio-banding in judo: The mediation role of anthropometric variables on the maturation effect. Int. J. Environ. Res. Public Health.

[49] Kato S., Yamagiwa S. (2021). Statistical extraction method for revealing key factors from posture before initiating successful throwing technique in judo. Sensors.

[50] Kato S., Yamagiwa S. (2022). Predicting successful throwing technique in judo from factors of kumite posture based on a machine-learning approach. Computation.

[51] Breen M., Reed T., Breen H.M., Osborne C.T., Breen M.S. (2022). Integrating wearable sensors and video to determine microlocation-specific physiologic and motion biometrics-method development for competitive climbing. Sensors.

[52] Blanco Ortega A., Isidro Godoy J., Szwedowicz Wasik D.S., Martínez Rayón E., Cortés García C., Ramón Azcaray Rivera H., Gómez Becerra F.A. (2022). Biomechanics of the upper limbs: A review in the sports combat ambit highlighting wearable sensors. Sensors.

[53] Kostrzewa M., Laskowski R., Wilk M., Błach W., Ignatjeva A., Nitychoruk M. (2020). Significant predictors of sports performance in elite men judo athletes based on multidimensional regression models. Int. J. Environ. Res. Public Health.

[54] Ouergui I., Delleli S., Chtourou H., Selmi O., Bouassida A., Bouhlel E., Franchini E. (2022). Diurnal Variation of specific tests’ performance and related psychological aspects in young judo athletes. Res. Q. Exerc. Sport.

[55] Ambrozy T., Cynarski W.J., Czarny W., Błach W., Cetini´c M.C., Dukari´cdukari´c V., Segedi I., Rupči´c T.R., Serti´c H.S. (2022). Defining the influence of fatigue protocol on kinematic parameters of ippon seoi nage. Appl. Sci..

[56] Pucsok J.M., Nelson K., Ng E.D. (2001). A Kinetic and kinematic analysis of the harai-goshi judo technique. Acta Physiol. Hung..

[57] Imamura R., Johnson B. (2003). A kinematic analysis of a judo leg sweep: Major outer leg reap-osoto-gari. Sports Biomech..

